# Surfactant Protein A Impairs Genital HPV16 Pseudovirus Infection by Innate Immune Cell Activation in A Murine Model

**DOI:** 10.3390/pathogens8040288

**Published:** 2019-12-06

**Authors:** Sylvia Ujma, Sinead Carse, Alisha Chetty, William Horsnell, Howard Clark, Jens Madsen, Rose-Marie Mackay, Alastair Watson, Mark Griffiths, Arieh A. Katz, Georgia Schäfer

**Affiliations:** 1 Division of Medical Biochemistry and Structural Biology, Department of Integrative Biomedical Sciences, University of Cape Town, Cape Town 7925, South Africa; sylvia.ujma@live.com (S.U.); carsesinead@gmail.com (S.C.); arieh.katz@uct.ac.za (A.A.K.); 2 Division of Immunology, Department of Pathology, University of Cape Town, Cape Town 7925, South Africa; allychetty@gmail.com (A.C.); wghorsnell@gmail.com (W.H.); 3 Institute of Infectious Disease and Molecular Medicine, University of Cape Town, Cape Town 7925, South Africa; 4 Institute of Microbiology and Infection, University of Birmingham, Birmingham B15 2TT, UK; 5 Institute for Women’s Health, University College London, London WC1E 6BT, UK; howard.clark@me.com (H.C.); J.Madsen@soton.ac.uk (J.M.); 6 Child Health, Division of Clinical and Experimental Sciences, Department of Child Health, University of Southampton, Southampton SO17 1BJ, UK; R.A.Mackay@soton.ac.uk (R.-M.M.); a.s.watson@soton.ac.uk (A.W.); 7 National Heart & Lung Institute, Imperial College London, London SW7 2AZ, UK; m.griffiths@imperial.ac.uk; 8 SAMRC/UCT Gynaecological Cancer Research Centre (GCRC), Cape Town 7925, South Africa

**Keywords:** human papillomavirus (HPV), surfactant proteins A and D (SP-A and SP-D), murine cervicovaginal challenge model, innate immune cells, opsonins

## Abstract

Infection by oncogenic human papillomavirus (HPV) is the principle cause of cervical cancer and other anogenital cancers. The majority of cervical cancer cases occur in low- and middle-income countries (LMIC). Prophylactic vaccines exist to combat HPV infection but accessibility to these in LMIC is limited. Alternative preventative measures against HPV infection are therefore also needed to control cervical cancer risk. HPV employs multiple mechanisms to evade the host immune response. Therefore, an approach to promote HPV recognition by the immune system can reduce infection. Surfactant proteins A and D (SP-A and SP-D) are highly effective innate opsonins of pathogens. Their function is primarily understood in the lung, but they are also expressed at other sites of the body, including the female reproductive tract (FRT). We hypothesized that raised levels of SP-A and/or SP-D may enhance immune recognition of HPV and reduce infection. Co-immunoprecipitation and flow cytometry experiments showed that purified human SP-A protein directly bound HPV16 pseudovirions (HPV16-PsVs), and the resulting HPV16-PsVs/SP-A complex enhanced uptake of HPV16-PsVs by RAW264.7 murine macrophages. In contrast, a recombinant fragment of human SP-D bound HPV16-PsVs weakly and had no effect on viral uptake. To assess if SP-A modulates HPV16-PsVs infection in vivo, a murine cervicovaginal challenge model was applied. Surprisingly, neither naïve nor C57BL/6 mice challenged with HPV16-PsVs expressed SP-A in the FRT. However, pre-incubation of HPV16-PsVs with purified human SP-A at a 1:10 (w/w) ratio significantly reduced the level of HPV16-PsV infection. When isolated cells from FRTs of naïve C57BL/6 mice were incubated with HPV16-PsVs and stained for selected innate immune cell populations by flow cytometry, significant increases in HPV16-PsVs uptake by eosinophils, neutrophils, monocytes, and macrophages were observed over time using SP-A-pre-adsorbed virions compared to control particles. This study is the first to describe a biochemical and functional association of HPV16 virions with the innate immune molecule SP-A. We show that SP-A impairs HPV16-PsVs infection and propose that SP-A is a potential candidate for use in topical microbicides which provide protection against new HPV infections.

## 1. Introduction

Human papillomavirus (HPV) is the most common viral infection of the reproductive tract. According to their association with malignancy, HPVs are broadly divided into high-risk and low-risk types. High-risk HPVs represent one of the seven groups of oncogenic viruses known to date which have been recognized to be consistently associated with various types of human cancer [1], with HPV types 16 and 18 being the most common oncogenic HPV types world-wide and the major cause of cervical cancer [2]. This disease is substantially more prevalent in low-and middle-income countries (LMIC), and is the leading cause of female cancer-associated deaths in Sub-Saharan Africa, not least due to the concomitant Human immunodeficiency virus (HIV)/acquired immunodeficiency syndrome (AIDS) epidemic in this region [3,4,5]. Although highly efficient prophylactic vaccines against the most common high-risk HPV types exist, roll out to LMIC has been severely limited due to cost, difficulties in reaching the target population, cultural challenges, and the necessity for a cold chain [6]. Therefore, alternative means to protect against sexually transmitted HPVs are much needed. For example, the naturally-derived sulphated polysaccharide carrageenan has been shown to inhibit early HPV infection by blocking viral attachment to cells [7,8]. This inhibitory effect was supported by clinical trials demonstrating a negative association of HPV infection with the vaginal microbicide Carraguard [9]. However, while carrageenan also showed protection against herpes simplex virus 2 (HSV-2) [10], it did not prevent against vaginal transmission of HIV [11].

Another alternative approach for prevention of HPV infection is the activation of the innate immune response leading to enhanced recognition and elimination of the virus before infection of its target cells. Most HPV infections are transient with clearance mediated by cell-mediated immune mechanisms usually within 6–12 months, leaving the infected individual partly or fully immune to that particular HPV type. However, failure to develop effective immune responses and depending on HPV type, viral load, immune status of the host and/or ongoing HPV exposure, the virus can reach the squamous epithelial cells where it remains effectively ‘hidden’ from the host immune system resulting in persistent infection and an increased probability of progression into invasive cancers [12]. Indeed, HPV employs several mechanisms to effectively evade the innate immune response thereby delaying the activation of adaptive immunity as the virus is not cytolytic, there is no viremia, no inflammation and no danger signals to alert the immune system [12]. 

Surfactant proteins (SPs), particularly SP-A and SP-D, are innate immune molecules which enhance pathogen recognition and phagocytosis by opsinization [13]. They were originally identified in the lung as components of pulmonary surfactant involved in maintaining lung homeostasis and protecting it from infection. Both proteins are therefore best studied for their roles in the control of various respiratory pathogens, including influenza A virus, respiratory syncytial virus, *Mycobacterium tuberculosis*, *Aspergillus fumigatis*, *Pseudomonas aeruginosa*, *Haemophilus influenza*, and *Nippostrongylus brasiliensis* [14,15,16,17,18,19,20,21,22]. However, their expression at various non-pulmonary sites, including the female reproductive tract (FRT), suggests additional yet largely unexplored roles for pathogen control in these compartments [23]. 

SP-A and SP-D are members of the collectins family. They are large hydrophilic soluble proteins that act as collagenous C-type lectin pattern recognition receptors, generally in a calcium dependent manner [24]. Their primary structure is composed of an N-terminal non-collagenous domain which can form inter-subunit disulphide bonds, followed by a collagenous region of Gly-X-Y repeats, a helical neck domain and a globular C-terminal carbohydrate recognition domain (CRD). Trimers are formed by spontaneous self-assembly of such monomeric units which further form higher-order bunch-like SP-A octadecamers and cruciform SP-D dodecamers, respectively [25]. Despite their similar domain architectures, SP-A and SP-D differ in their ligand binding specificities. SP-A preferentially binds to mannose, fucose, and lipid ligands on the surface of incoming pathogens, while SP-D mostly binds maltose, inositol, glucose and more complex carbohydrates [26,27]. Both SP-A and SP-D have very low affinities to galactose and sialic acid (sugars that often form the terminals of carbohydrates on animal cells) which is important for distinguishing self from non-self [27]. SPs recognition and binding of their specific ligands often occurs via their CRDs and triggers various immune responses, including opsonization and enhanced phagocytosis, regulating macrophage function and inflammation, and killing [27]. In the human FRT, SP-A has been detected in the myometrium, vaginal epithelium, and vaginal lavage fluid, while SP-D was found in the cervix, vagina, and endometrium [28,29,30]. It has been suggested that both SPs play protective roles during pregnancy [31] as well as contribute to innate immune defences against sexually transmitted pathogens [32,33,34]. 

In an attempt to identify novel molecules that enhance immune recognition of oncogenic HPV, we herein demonstrate for the first time that SP-A but not SP-D binds directly to HPV16-PsVs and increases viral uptake into innate immune cells, thereby attenuating overall cervicovaginal infection in a mouse model.

## 2. Results

### 2.1. Binding of HPV16-PsVs to SP-A but Not SP-D Results in Increased Viral Uptake by RAW264.7 Macrophages

**Figure 1:**
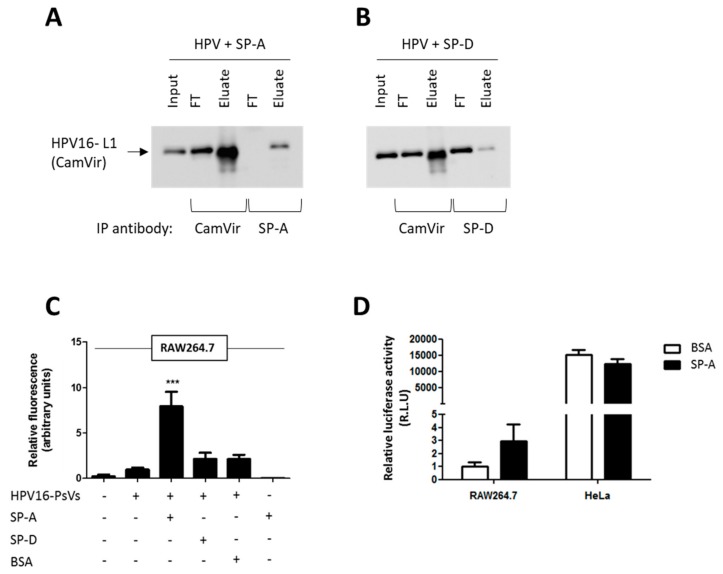
Binding of HPV16-PsVs to SP-A but not SP-D results in increased viral uptake by RAW264.7 macrophages but not by HeLa cervical epithelial cells. (**A**,**B**) Co-immunoprecipitation experiments displaying the input, flow through (FT) and eluate samples of HPV16-PsVs incubated with (A) SP-A and (B) SP-D. Complexes were incubated for 1h at 4 °C, followed by precipitations with either anti-HPV16-L1 (CamVir), anti-SP-A or anti-SP-D antibodies. Western blots were performed using the CamVir antibody to detect the presence of HPV16-L1. (**C**) RAW264.7 cells were infected with fluorescently labelled HPV16-PsVs (pre-absorbed with recombinant proteins where indicated) for 1 h at 37 °C. Cells were washed extensively and lifted with trypsin/EDTA to remove surface-bound virions and subjected to flow cytometry to quantify viral internalization. Experiments were performed in triplicates, quantified by quadrant analysis of the dot blots of three independent experiments and presented as x-fold increase relative to the mean fluorescence intensity of cells infected with untreated HPV16-PsVs which was set as 1. Significances were calculated by means of one-way ANOVA and Tukey post-hoc tests. *** indicates statistical significance between uptake of HPV16-PsVs in the presence of SP-A as compared to the other tested conditions (*** = *p* < 0.001). (**D**) RAW264.7 and HeLa were infected with HPV16-PsVs encapsidating the firefly luciferase reporter plasmid pGL3-control for 48 h. Where indicated, the viral particles were pre-absorbed with purified SP-A protein (or BSA control) before infection. Firefly luciferase activities in the cell lysates as a measure for successful infection is presented as Relative Light Units relative to RAW264.7 cells infected with untreated HPV16-PsVs which was set as 1. Bars in C and D depict mean values, with error bars showing SEM. Surfactant proteins A and D: SP-A, SP-D.

In an attempt to identify molecules that enhance innate immune recognition of oncogenic HPV thereby preventing initial infection, we studied the role of surfactant proteins A and D on HPV16 pseudovirion (HPV16-PsVs) infection. Co-immunoprecipitation experiments of purified human SP-A or recombinant SP-D together with HPV16-PsVs using antibodies against SP-A, SP-D and HPV16 L1 (CamVir) revealed a direct biochemical association between the virions and SP-A and to a much lesser degree with recombinant SP-D: while HPV16-PsVs could be detected in the eluate (but not the FT fraction) when the HPV/SP-A input was immunoprecipitated with an SP-A antibody (Figure 1A), the majority of the virions derived from the HPV/SP-D input were detected in the FT fraction and very little in the eluate when immunoprecipitated with an SP-D antibody (Figure 1B). To test whether this physical interaction between SP-A and HPV16-PsVs had any functional consequences on immune cell recognition, virus internalization assays using the murine macrophage cell line RAW264.7 were performed. As presented in Figure 1C, pre-incubation of the viral particles with SP-A, but not with SP-D, significantly enhanced their uptake by the macrophages (Figure 1C). We also determined downstream reporter gene activity 48h post-infection as a measure of successful infection, i.e., the transcription of the firefly luciferase plasmid encapsidated in the viral particle representing the pseudogenome. It was noted that the infectivity of RAW264.7 macrophages by HPV16-PsVs compared to the human cervical cancer cell line HeLa cells was extremely low (approximately 15,000-fold less, Figure 1D). However, when HPV16-PsVs were pre-incubated with SP-A, luciferase activity measured in RAW264.7 cells increased by approximately three-fold, but had no effect on HeLa cell infection (Figure 1D). We therefore focused our experiments on viral immune recognition and uptake and hypothesized that macrophages may prevent infection by effectively killing most of the internalized virus which may be facilitated by SP-A. This is supported by the general assumption that HPV has a very strict tissue tropism in vivo and exclusively infects basal epithelial cells [35]. To our knowledge, macrophages are not known to be infected by HPV.

### 2.2. SP-A-Mediated HPV16-PsVs Uptake by RAW264.7 Macrophages Is Calcium-Dependent, but Not Dependent on the Lectin-Binding Domain

**Figure 2:**
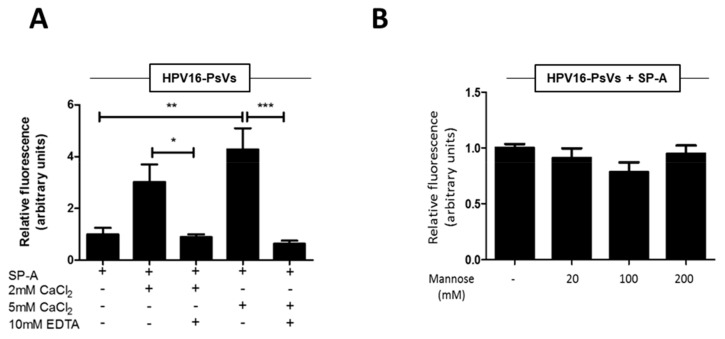
SP-A-mediated HPV16-PsVs uptake by RAW264.7 macrophages is calcium-dependent, but not dependent on the CRD. AF488-labelled HPV16-PsVs were incubated in the presence of 10-fold (w/w) excess SP-A and (**A**) with or without 2 mM or 5 mM calcium chloride in the presence or absence of 10 mM EDTA as indicated, or (**B**) various concentrations of mannose. Shown are summaries of viral internalisation experiments as determined by FACS as described in Figure 1C. Fluorescent readings are displayed relative to HPV16-PsVs internalisation in the presence of SP-A only which was set as 1. Quantification of three independent experiments performed in duplicate are shown. Statistical significance was determined using one-way ANOVA and Tukey post-hoc tests. * = *p* < 0.05, ** = *p* < 0.01, *** = *p* < 0.001. Bars depict mean values, with error bars showing SEM.

SP-A is known to recognize and bind specific carbohydrate moieties on the surface of microorganisms via their CRD. For example, it has been shown that SP-A targets the HIV envelope glycoprotein gp120 in a calcium-dependent manner, which could be inhibited by mannose and EDTA [32]. Although HPV is a non-enveloped virus not known to contain glycosylated proteins in its capsid, we set out to test whether this mode of interaction was also applicable to HPV16-PsVs. We therefore conducted further HPV16-PsVs internalization experiments with SP-A-pre-incubated virions in the absence or presence of CaCl_2_ or mannose. As presented in Figure 2A, viral uptake by RAW264.7 macrophages was strongly enhanced with increasing CaCl_2_ concentrations, an effect that was completely abolished in the presence of EDTA. However, viral uptake was not affected by mannose (Figure 2B) which is known to act as a competitive CRD inhibitor [36]. We therefore conclude that SP-A, as a typical C-type lectin, requires calcium for binding and functionality, while this does not involve its lectin-binding domain itself. 

### 2.3. Cervicovaginal Challenge with HPV16-PsVs Does Not Alter SP-A Expression in the Murine FRT 

**Figure 3:**
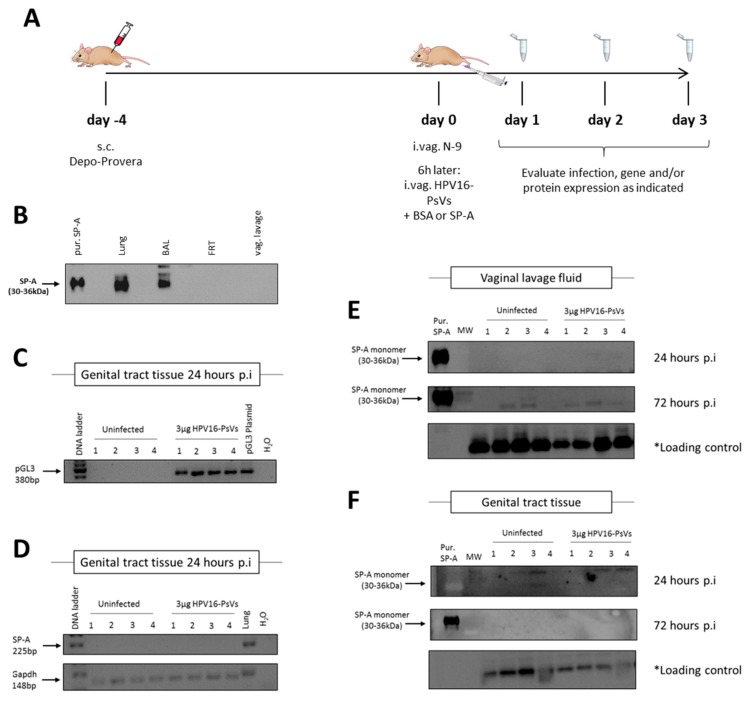
Infection of C57BL/6 mice with HPV16-PsVs does not alter SP-A expression. (**A**) Mouse model for HPV16-PsVs infection using C57BL/6 mice, adapted from Roberts et al., 2007 [8,37]. Briefly, 6–10 weeks old female wildtype C57/BL6 mice were injected with 2 mg Depo-Provera (s.c.) for 4 days, and then pre-treated with 25 µL 4% N-9 in 3% CMC i.vag. for 6 h prior to HPV16-PsVs infection. Six mice per group were i.vag. infected with 3 µg or 1 µg viral particles encapsidating the reporter gene firefly luciferase (pGL3-control) or Gaussia luciferase (pGLuc), respectively. After 1–3 days, tissues were harvested for analysis. (**B**) Western blot assessing SP-A expression in lung, bronchoalveolar lavage fluid (BAL), female reproductive tract (FRT) tissue, and vaginal lavage (vag. lavage) fluid of naïve wildtype C57BL/6 mice. 20 µg of each sample was loaded per lane, while 0. 5 µg of purified human SP-A protein was loaded as control. No endogenous SP-A was detected in the FRT of naïve mice. (**C**) Four mice per group were i.vag. infected with 3 µg HPV16-PsVs encapsidating the pGL3-control reporter plasmid. Genital tract tissue was harvested 24 h later and RNA extracted for gene expression analysis to confirm successful infection. (**D**) RNA from (**C**) was assessed for SP-A expression, with lung RNA used as positive control. Gapdh was used as a reference gene. (**E**) Four mice per group were i.vag. infected with 3 µg HPV16-PsVs. Vaginal lavages were performed 24 h and 72 h p.i. and assessed by Western Blot for the presence of SP-A. 20 µg of each sample was loaded per lane, while 0.5 µg purified human SP-A protein was loaded as control. * Loading control corresponds to a 45 kDa cellular protein that cross-reacts with the CamVir primary antibody [38]. (**F**) Four mice per group were i.vag. infected with 3 µg HPV16-PsVs and genital tract tissue was harvested 24 h or 72 h p.i., homogenised and assessed by Western blot for the presence of SP-A as described in (E).

While the in vitro findings described above suggest a role of SP-A in enhancing immune recognition of incoming HPV16-PsVs, the natural HPV infection environment in the genital tract is very complex as several diverse cell types are potentially involved in immune responses upon viral challenge, including local epithelial cells and recruited inflammatory cells. We therefore studied the impact of SP-A on HPV16-PsVs infection in a murine model which particularly reflects the early events in infection [8,37] (Figure 3A). 

To assess whether the level of SP-A in the murine FRT changes upon HPV challenge, we performed several gene and protein expression assays at 24 h and 72 h post infection. While SP-A is known to be expressed in the human FRT [30], to our surprise no SP-A was detected in either the vaginal lavage fluid or homogenized FRTs of naïve C57BL/6 mice (Figure 3B). Moreover, exposure to HPV16-PsVs neither induced SP-A gene expression in the murine FRT 24 h post infection (Figure 3C,D) nor did it increase detectable SP-A protein 24 h or 72 h post infection in the murine FRT or vaginal lavage fluid, respectively (Figure 3E,F). Therefore, infection with HPV16-PsVs does not affect endogenous SP-A expression in the FRT of C57BL/6 mice.

### 2.4. HPV16-PsVs Complexed with SP-A Reduces Infection by Increasing Macrophage Recruitment and Recognition by Innate Immune Cells

**Figure 4:**
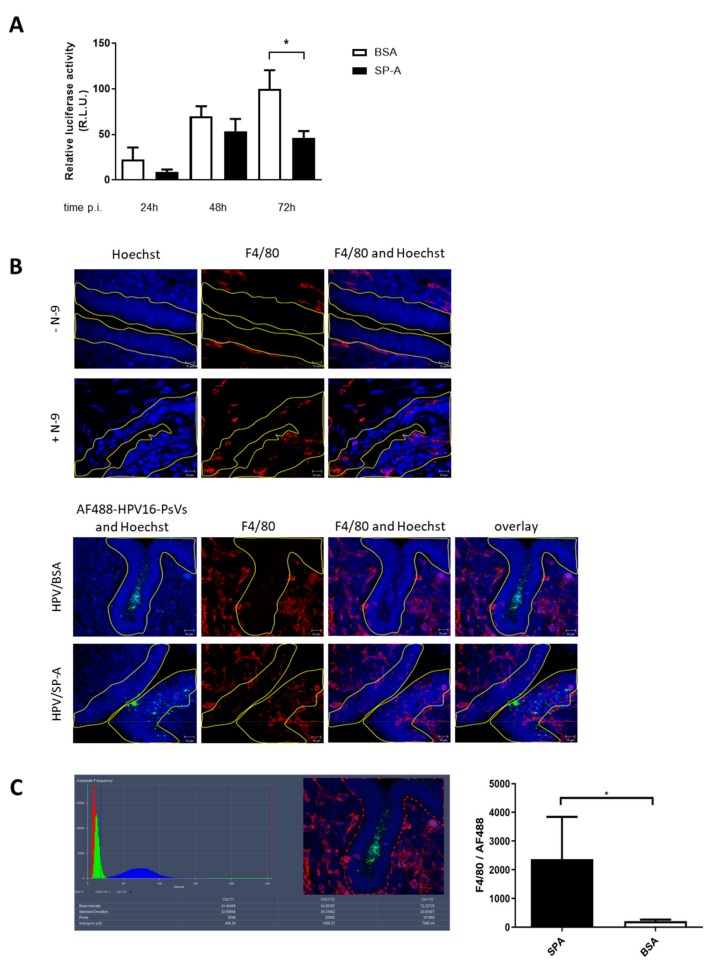
SP-A reduces HPV16-PsVs infection in C57BL/6 mice by increasing macrophage recruitment into the basal epithelium. 6–10 weeks old female wildtype C57BL/6 mice were pre-treated as described in Figure 3A. (**A**) HPV16-PsVs encapsidating the Gaussia luciferase reporter plasmid pGLuc were pre-incubated with a 10-fold (w/w) excess of purified SP-A protein (or BSA control) for 1 h at room temperature, before intravaginal inoculation. Genital tracts were washed with 2 × 50 µL PBS at 24 h, 48 h, and 72 h post infection, and activity of the secreted Gaussia luciferase in the vaginal lavage fluid as a measure for infection was determined. Data of three independent experiments are presented relative to infectivity of the BSA control group at 72 h which was set as 100%. Statistical significance was determined using one-way ANOVA and Bonferroni’s multiple comparison tests for the individual time points. *= *p* < 0.05. Bars depict mean values, with error bars showing SEM. (**B**) Mice were treated with N-9 for 6 h as required for HPV challenge (Figure 3A) or left untreated (upper panel). AF488 labelled HPV16-PsVs were pre-incubated with 10-fold (w/w) excess of purified SP-A protein (or BSA control) for 1 h at room temperature, before intravaginal inoculation (lower panel) or left uninfected (upper panel). Genital tracts were dissected 2 h post infection where indicated, embedded in OCT and cryosectioned, followed by staining for nuclei (Hoechst) and macrophages (F4/80). Representative confocal images of epithelial tissue (blue) exposed to fluorescent virions (green) is shown with staining for macrophages in red. The basal epithelium is outlined in yellow. (**C**) The area of F4/80 staining in the basal layer of the epithelium (outlined) of 10 randomly chosen images was quantified by setting the lower and upper thresholds at 18 and 256, respectively, to remove any background signal. Macrophage recruitment to the epithelium in response to SP-A coated HPV16-PsVs was compared to the BSA control group. Statistical significance was determined using a Mann-Whitney test. * = *p* < 0.05. Bars depict mean values, with error bars showing SEM.

Since SP-A was not detectable in the FRT of both naïve and HPV16-challenged C57BL/6 mice, we asked whether artificial supplementation with exogenous SP-A had an effect on HPV infection. As shown in Figure 4A, pre-incubation of HPV16-PsVs with purified SP-A at a 1:10 (w/w) ratio decreased the level of infection over time with significant reduction seen 72 h post infection. 

To determine whether the presence of SP-A elicited an immune response that led to the observed overall decrease in HPV16-PsVs infection, C57BL/6 mice were challenged with SP-A-complexed AF488-conjugated HPV16-PsVs for 2 h, followed by FRT dissection and preparation for macrophage staining and visualization by confocal microscopy. Representative images are shown in Figure 4B (lower panel) which demonstrate macrophage recruitment into the basal epithelium and to the site of infection in the presence of SP-A but not BSA control. However, it should be noted that there was an overall recruitment of macrophages into the FRT due to N-9 treatment prior to viral challenge which was independent of HPV (Figure 4B, upper panel). It is a well-known phenomenon that N-9 elicits a strong inflammatory response [39]; therefore, we quantified macrophage recruitment into the basal epithelium by comparing infectious conditions with SP-A-coated virions with BSA controls only. Indeed, quantification of the area occupied by macrophages (normalized to the amount of SP-A/or BSA-pre-treated virus present) of 10 randomly chosen images further indicated a significant increase in macrophage recruitment into the basal epithelium in the presence of SP-A. 

**Figure 5:**
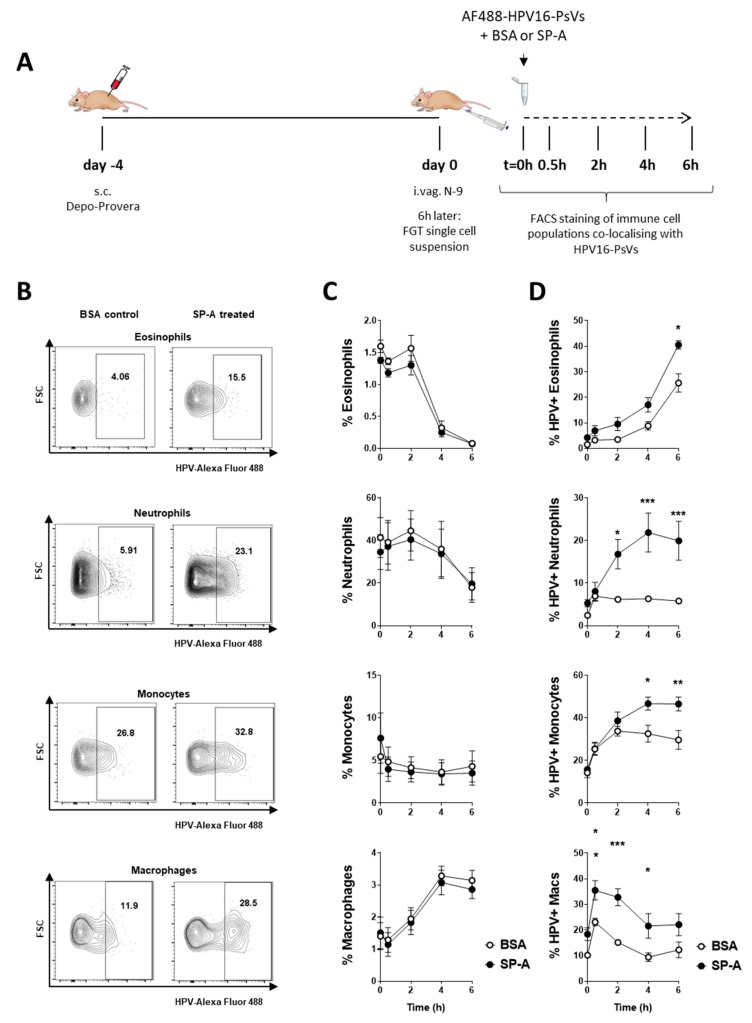
Ex vivo infection of FRT single cell suspension with HPV16-PsVs pre-treated with SP-A leads to increased viral uptake by selected immune cell populations. (**A**) 6–10 weeks old female wildtype C57/BL6 mice were treated with Depo-Provera (s.c.) for four days, and then pre-treated with N-9 for 6 h. Mice were killed and FRTs dissected for the preparation of single cell suspensions. Cells were infected with AF488-labelled HPV16-PsVs that had been preincubated with BSA (control) or SP-A in a ratio of 1:10 (w/w) for 1 h at room temperature. At the indicated time points, cells were stained to visualize and gate the following immune cell populations by flow cytometry: eosinophils (CD11b^+^/SiglecF^+^/SSC^high^), neutrophils (CD11b^+^/Gr-1^high^), inflammatory monocytes (CD11b^+^/Gr-1 ^intermediate^), and macrophages (CD11b^+^/F4/80^+^). (**B**) Representative flow gates of AF488-labelled HPV16-PsVs uptake/co-localisation by innate genital cells at the 2 h time point are shown. (**C**) Shown is the percentage of the indicated immune cell populations over time upon infection with HPV16-PsVs pre-incubated with BSA (control) or SP-A. Pooled data of two independent experiments are presented. (**D**) Shown is the uptake of HPV16-PsVs with the indicated immune cell populations over time dependent on pre-incubation of the viral particles with BSA (control) or SP-A. Pooled data of two independent experiments are presented. Significances were calculated by means of two-way ANOVA and Bonferroni’s multiple comparison tests for the individual time points, with * = *p* < 0.05; ** = *p* < 0.01; *** = *p* < 0.001. Depicted are mean values, with error bars showing SEM.

To overcome the limitations of quantifying microscopy images and to further demonstrate innate immune cell recognition of HPV16-PsVs complexed with SP-A, we isolated FRT cells from naïve C57BL/6 mice, which were then exposed to AF488-conjugated HPV16-PsVs ex vivo over a 0–6 h time course (Figure 5A). Isolated genital cells were analyzed for eosinophils, neutrophils, monocytes, and macrophages by multi-color flow cytometry. Innate cell gating is outlined in Supplementary Figure S1. While the number of these innate immune cell populations did not change dependent on SP-A over time (Figure 5C), co-localization with AF488-conjugated HPV16-PsVs significantly increased in the presence of SP-A for all studied innate cell populations (Figure 5D). This is exemplified for the 2 h time point in Figure 5B.

In summary, we conclude that the SP-A mediated uptake of HPV16-PsVs by various innate immune cell populations impairs the overall establishment of infection. 

## 3. Discussion

As multifunctional host defense collectins, surfactant proteins A and D act as a pattern recognition receptors, identifying and binding to invading pathogens via their signature pathogen-associated molecular patterns, leading to opsonization, recruitment and activation of innate immune cells, enhanced phagocytosis (by increasing cell-surface expression of receptors involved in phagocytosis), and killing via oxidative mechanisms [27]. SP-A and SP-D therefore function as a first line of defense against invading pathogens before the development of specific antimicrobial humoral responses.

While diverse respiratory pathogens have been shown to be controlled by SP-A and SP-D, recent reports suggested a protective role of these proteins at non-pulmonary sites such as the FRT [23]. We therefore set out to determine whether HPV, the most common sexually transmitted virus, is recognized and modulated by SP-A and/or SP-D. We demonstrated by biochemical pull-down experiments that SP-A (and to a much lesser extent SP-D) bound directly to HPV16-PsVs. It is important to note, however, that the native SP-A protein used for these assays consisted of six trimeric units (hence has 18 binding sites per oligomerised molecule and a potentially high avidity) while the recombinant trimeric fragment of SP-D consisted only of three binding sites and has therefore potentially lower avidity. The involvement of SP-D in HPV binding should therefore be investigated in the future with the native protein.

Consequently, only binding to SP-A had functional consequences for enhanced immune cell recognition as demonstrated by increased viral uptake into RAW264.7 murine macrophages. Similar observations have been reported for other pathogens found in the reproductive tract: artificial supplementation with SP-A protein increased phagocytosis of *Ureaplasma* by RAW264.7 murine macrophages [40] and *Chlamydia* by THP-1 human macrophages [41].

SPs are known to exert their function in a calcium-dependent manner, and crystallographic and solution binding studies have elucidated SP-A’s calcium-binding sites, with one being located in the CRD, whilst another site is located in an anionic portion of the neck region [42]. We confirmed a calcium-dependent increase of HPV16-PsVs uptake into RAW264.7 murine macrophages in the presence of SP-A and hypothesize that the calcium binding site in the neck region is important in this context as the lectin-binding domain in the CRD seems to not be involved in binding and increased viral internalisation as revealed by competition studies using mannose. This is in stark contrast to SP-A’s mode of binding to HIV, for example: here, SP-A has been reported to bind to HIV’s envelope glycoprotein gp120 via its CRD in a calcium-dependent manner that is inhibitable by mannose and EDTA [32]. The untypical C-type lectin activity of SP-A in the context of HPV16-PsVs is not surprising as HPV is a non-enveloped virus consisting of a protein capsid not known to display glycosylated surface/capsid proteins. SP-A may instead recognize HPV16-PsVs in an alternate manner, such as via the neck domain. Current work in our laboratory focuses on the identification of the specific ligand binding site on HPV16-PsVs.

To assess the impact of SP-A during the early events of sexual HPV transmission, we studied infection in a murine cervicovaginal challenge model using wildtype C57BL/6 mice. Although SP-A has been reported to be expressed in the human vaginal mucosa and vaginal lavage fluid [30], to our surprise SP-A was not detected in the murine FRT, neither with nor without HPV16-PsVs challenge. This is opposed to the murine urinary tract where the presence of SP-A has recently been described [43]. While SP-D has been reported to be expressed in the murine FRT [44], to our knowledge, no studies on SP-A in this compartment have been published yet. 

The absence of SP-A in the murine FRT allowed us to study the effect of artificial supplementation with exogenous SP-A on HPV16-PsVs challenge. Pre-incubation of the virions with a 10-fold excess (w/w) human SP-A protein led to an overall decrease in HPV16-PsVs infection, possibly due to increased SP-A-mediated immune cell recognition and elimination of infectious virions. Indeed, by confocal microscopy we observed an increased amount of macrophages at the site of infection in the presence of SP-A. Similar results have recently been reported for uropathogenic *Escherichia coli*: supplementation with SP-A (but not SP-D) resulted in decreased bacterial growth in the murine urinary tract, concurrent with a considerable number of infiltrated immune cells [43]. 

To study selected innate immune cell populations for their ability to take up HPV16-PsVs in the presence or absence of SP-A, single cell suspensions prepared from murine FRT tissue were analyzed by flow cytometry over a time course of 6 h. All assessed populations, i.e. eosinophils, neutrophils, monocytes, and macrophages displayed strong SP-A-mediated viral uptake compared to control, with neutrophils being the most significant cellular responders to HPV16-PsVs infection in the presence of SP-A.

Based on our murine model, these data suggest an SP-A-mediated opsonization of incoming HPV16-PsVs, increased phagocytosis and killing by innate immune cells with an overall effect on dampening the infection. Besides enhanced phagocytosis, SPs are also known to aggregate pathogens thereby hindering their entry into host cells. Although we did not study this effect, the inhibitory effect of SP-A is not necessarily dependent on agglutination [43], and our data strongly point toward SP-A-mediated innate immune cell uptake of the virions. While we demonstrated direct biochemical interaction between HPV16-PsVs and SP-A, suggesting opsonization as causal basis for enhanced phagocytosis, we cannot exclude that the mere presence of exogenously supplied SP-A in the murine FRT contributed to enhanced infiltration of innate immune cells to the site of infection and their prompt clearance of the invading viral particles. 

Our findings provide a strong basis that encourage further studies into possible broad-spectrum activities of SP-A in the prevention of initial oncogenic HPV infection and/or viral shedding in the genital tract. While this study focused on HPV type 16, the most common oncogenic HPV type worldwide [2], it is likely that SP-A also recognizes other HPV types, both low-risk and high-risk. Indeed, innate immune molecules, such as opsonins, generally have a broad range of pathogen recognition and therefore could potentially be protective not only against HPV but also other sexually transmitted pathogens. For example, SP-A was shown to bind to HIV, thereby protecting CD4^+^ cells from direct infection [32]. However, SP-A plays a dual role in this scenario as it enhances dendritic cell-mediated transfer of HIV in vitro [32]. Studies on the role of SPs during pregnancy suggested that SP-A and SP-D had a protective function in the murine decidua; and it was proposed that exogenous administration of SP-A (and SP-D) could potentially control infection during spontaneous term labor and infection-induced preterm labor [45].

While our study showed no endogenous SP-A expression in the murine FRT, SP-A has previously been detected in the human vaginal mucosal tissue [30]. However, the amount of SP-A detected was substantially less than what is typically found in the lung where SP-A is well known to play an important role in innate immune defence. It is therefore not clear whether the amount of SP-A in the human FRT is sufficient to have an impact on efficient pathogen recognition. Since HPV is known to efficiently evade innate immune recognition and infect its host relatively “unrecognized”, it can be assumed that natural endogenous levels of SP-A do not substantially affect HPV infection in humans and that SP-A levels comparable to the lung are needed for efficient virus elimination. This might possibly explain why respiratory HPV infection is a very rare condition [46], although no studies investigating the association between SP-A levels and pulmonary HPV infection in patient with recurrent respiratory papillomatosis have been conducted to our knowledge.

In summary, this study assigned a novel role for SP-A in the protection against HPV16-PsVs infection in the murine FRT. Future research aims to develop this molecule into a topical microbicide as an alternative and potentially broad-spectrum means to current prophylactic HPV vaccination.

## 4. Materials and Methods

### 4.1. Cell Culture

The murine leukemic macrophage cell line RAW264.7 (ATCC^®^ #TIB-71™), the human epithelial cervical adenocarcinoma cell line HeLa (ATCC^®^ #CCL-2™) and the virus packaging cell line HEK293TT (established from primary embryonal human kidney cells transformed with modified human adenovirus) [47] were grown and maintained in DMEM (Life Technologies, Carlsbad, CA, USA) supplemented with 10% heat-inactivated fetal calf serum (Biochrom, Cambridge, UK), 100 U/ml penicillin and 100μg/ml streptomycin (complete DMEM). All cells were grown at 37 °C in 5% CO_2_/95% air humidified atmosphere.

### 4.2. Purification of Native Human SP-A and Recombinant SP-D Proteins

Human SP-A was purified from bronchoalveolar lavage fluid (BAL) from human patients with alveolar proteinosis using a butanol extraction method, as previously described [48]. The BAL was collected from patients with informed consent and the necessary ethical permission (the Royal Brompton and Harefield Research Ethics Committee NRES10/H0504/9). The patients underwent the procedure for therapeutic purposes. The recombinant fragment of human SP-D (rfhSP-D), a recombinant trimeric neck/CRD fragment of SP-D, including a short region of the collagen stalk (8 Gly-X-Y) and representing residues 179 to 355, was purified using a bacterial expression system as previously described [49]. The purified rfhSP-D protein was treated for endotoxin by addition of 1/5th of the protein sample volume of pre-washed Polymyxin B-Agarose beads according to manufactures protocol (Merck, Kenilworth, NJ, USA). Levels of endotoxin in the purified SP-A and rfhSP-D protein preparation was determined using a Limulus Amebocyte Lysate (LAL) chromogenic assay (Lonza, Basel, Seitzerland). An endotoxin level of 10 pg/µg of rfhSP-D protein was judged acceptable for use in cell based assays as described previously [33]. The butanol extracted SP-A had low endotoxin levels (<1 pg/μg) and was not endotoxin treated. The treated proteins were filtered using a 0.22 μm filter and stored as aliquots at −20 °C until usage. 

### 4.3. HPV16 Pseudovirion Preparation, Viral Internalisation and Infection Assays

HPV16-PsVs encapsidating either the intracellularly expressed firefly luciferase reporter gene plasmid pGL3-control (Promega, Madison, WI, USA) or the secreted Gaussia luciferase reporter gene plasmid pCMV-GLuc2 (New England Biolabs, Ipswich, MA, USA) were produced in 293TT cells by co-transfection with the plasmid pXULL which encodes codon-optimised HPV16 L1 and L2 following published procedures and quality controls [47,50]. Where indicated, the virions were labelled with Alexa Fluor^®^ 488 succinimidyl ester (AF488, Life Technologies, Carlsbad, CA, USA) before purification by CsCl density gradient centrifugation as described [51]. 

For viral internalisation studies, RAW264.7 cells were seeded in 6-well plates at a density of 5 × 10^5^ cells per well and grown overnight. AF488-labelled HPV16-PsVs were incubated with purified human SP-A or recombinant SP-D protein or BSA (Sigma, St. Louis, MO, USA) at a w/w ratio of 1:10 for 1 h at room temperature before addition to the cells at a final viral density of approximately 4 pg/cell for 1 h at 37 °C. Where indicated, SP-A was preincubated with increasing mannose concentrations (20–200 mM) or 2 mM and 5 mM CaCl_2_ (with or without 10 mM EDTA) for 1 h at 37 °C before addition of the virions. Cells were rinsed with PBS and harvested with 0.025% trypsin/0.01% EDTA in PBS to remove surface-bound virions, thereby allowing detection of internalized AF488-labelled viral particles by flow cytometry [50]. Cells were washed in FACS wash solution (0.5% BSA in PBS) and fixed with 1% (v/v) formaldehyde (Sigma) in PBS. The detection of AF488-positive cells was performed using a FACSCalibur (Becton Dickinson, Franklin Lakes, NJ, USA) together with the software CellQuest Pro. Quadrant statistics of three independent experiments performed in triplicate were used to calculate the means ± SEM. 

For infection studies, reporter gene expression was determined. RAW264.7 or HeLa cells were seeded in 12-well plates at a density of 1 × 10^5^ or 5 × 10^4^, respectively, cells per well and grown overnight. HPV16-PsVs encapsidating the plasmid pGL3-control were incubated with purified human SP-A protein or BSA at a w/w ratio of 1:10 for 1h at room temperature before addition to the cells at a final viral density of approximately 2 pg/cell at 37 °C for 48 h. Cells were washed with PBS, harvested and luciferase activity measured using the Luciferase Assay System kit (Promega) with the Fluoroscan Ascent FL (Thermo Fisher Scientific, Waltham, MA, USA). Raw luciferase data were normalised against total protein concentration of the lysates determined with the Pierce^™^ BCA Protein Assay Kit (Thermo Scientific, Waltham, MA, USA). Three independent experiments were performed in triplicate and used to calculate the means ± SEM.

For all internalization and infection experiments, one-way ANOVA and Tukey post-hoc tests were used for determination of statistical significance compared to controls.

### 4.4. Co-Immunoprecipitation and Western Blotting

To biochemically determine whether SP-A and/or SP-D interact with HPV16-PsVs, Co-IP experiments were performed by incubating either 5μg purified human SP-A or human SP-D with 0.2 μg HPV16-PsVs (or alone as a control) in complete DMEM for 1 h at 4 °C. Lysis/Wash Buffer (Thermo Scientific) was added to bring each solution’s total volume to 150μL. 5μL of each “input” samples were kept aside for analysis. 2.5 μg antibody (either CamVir1 against HPV16-L1 (Abcam, Cambridge, UK), mouse anti-human SP-A (Hyb 238-04, ThermoFisher (Waltham, MA, USA)) or rabbit anti-human SP-D [33] was then added to the appropriate samples and incubated overnight at 4 °C rotating. 25 μL Protein G Sepharose beads (Sigma) were added to each reaction, and tubes were incubated for 2 h at 4 °C rotating. Samples were added to spin columns (Thermo Scientific), and flow through (FT) collected by centrifugation at 1000 g for 1 min. Samples were washed three times with 200 μL Lysis/Wash Buffer, once with 100 μL 1× Conditioning Buffer (Thermo Scientific) and eluted with a total of 40 μL Elution Buffer (Thermo Scientific). Input, FT (3 μL each) and eluate samples (20 μL) were analysed by reduced SDS-PAGE followed by Western Blotting according to conventional protocols. Proteins were detected using the CamVir1 primary antibody in a 1:2500 dilution together with a goat-anti-mouse secondary antibody conjugated to horseradish peroxidase (HRP) (Bio-Rad, Hercules, CA, USA) in a 1:5000 dilution.

For detection of SP-A in mouse tissues, a rabbit anti-mouse SP-A antibody raised again a recombinant fragment of mouse SP-A was used at a 1:1000 dilution, followed by an anti-rabbit HRP secondary antibody (Jackson Laboratories, Bar Harbor, ME, USA).

All Western blots were visualised using the Lumi-Glo chemiluminescent substrate (KPL (seracare), Milford, MA, USA) together with a Biospectrum™ 500 Imaging System (Ultra Violet Products, UVP, Mile End South, SA, Australia).

### 4.5. HPV16-PsVs Murine Cervicovaginal Challenge Model

In order to recapitulate the early events of human sexual HPV transmission, a murine genital challenge model was applied as described before [8,37] (Figure 3A). Briefly, 4 days prior HPV infection, 6–10 weeks old C57BL/6 mice were treated systemically with progesterone by sub-cutaneous injection of 2 mg/20 g Depo-Provera (Pfizer) suspended in 200 µL PBS in order to equilibrate hormonal levels and facilitate infection of sexually transmitted diseases by prolonged diestrus [52]. 6 h prior HPV infection, mice were lightly anaesthetized by intra-peritoneal injection of 125 µL ketamine (1.5 mg/20 g) and xylazine (0.2 mg/20 g) and intravaginally received 25 µL of 4% Nonoxynol-9 (N-9) (abcam) in a formulation of 3% carboxymethylcellulose (CMC) in order to chemically disrupt the genital epithelial and to promote HPV infection [8]. For HPV infections, mice were again lightly anaesthetized and intravaginally inoculated with 1 µg HPV16-PsVs encapsidating the plasmid pCMV-GLuc2 or 3 µg virus encapsidating the plasmid pGL3-control in a total volume of 20 µL in the presence of 3% CMC formulation. Where indicated, viral particles were incubated with purified human SP-A protein or BSA at a w/w ratio of 1:10 for 1 h at room temperature before addition of CMC and intravaginal administration. Vaginal lavages were performed at 24 h, 48 h, and 72 h as indicated by rinsing the genital tract with 2× 50 µL sterile PBS. Cells and debris were removed by centrifugation and the remaining supernatants were analyzed for secreted Gaussia luciferase expression as a measure for infection using the Gaussia Luciferase Assay Kit (New England Biolabs) with the Fluoroscan Ascent FL (Thermo Fisher Scientific). Where indicated, mice were killed 24 h or 72 h post infection using halothane inhalation, and FRTs (excluding uterine horns and ovaries) were excised, snap frozen in liquid nitrogen and homogenised on ice in the presence of 500 μL 1× Cell Culture Lysis Reagent (Promega) or in the presence of 500 μL TRIzol™ reagent (Life Technologies) for further protein or gene expression analyses, respectively. 

All animal work was approved by the Faculty of Health Sciences Animal Ethics Committee, University of Cape Town (AEC 016/008).

### 4.6. Gene Expression Analysis

RNA was extracted from uninfected murine tissue (lung and FRT) and HPV16-PsVs challenged murine tissue (FRT) and reverse transcribed using the High-Capacity cDNA Reverse Transcription Kit (Applied Biosystems™, Foster City, CA, USA). Briefly, 1 μg total RNA was added to 0.8 μL 100 mM dNTP mix, 2 μL 10× RT Random Primers, 2 μL 10× RT Buffer, 1 μL MultiScribe™ Reverse Transcriptase (50 U/μL) and 1 μL RNase Inhibitor in a total volume of 20 μL. cDNA synthesis was performed by incubating the samples for 10 min at 25 °C, followed by 120 min at 37 °C and a final 5 min 85 °C denaturation. The cDNA was then used in subsequent PCRs to determine expression of SP-A, pGL3-control and GAPDH using the following primer pairs: SP-A forw ACCTGGATGAGGAGCTTAGACTGC and SP-A rev TGCTTGCGATGGCCTCGTTCT; pGL3 forw CGGGCGCGGTCGGTAAAGT and pGL3 rev AACAACGGCGGCGGGAAGT; GAPDH forw CCAATGTGTCCGTCGTGGATCT and GAPDH rev GTTGAAGTCGCAGGAGACAACC. Each reaction mixture contained 2.5 μL 10× DreamTaq Buffer (Fermentas, Waltham, MA, USA), 1 μL 10 μM forward primer, 1 μL 10 μM reverse primer, 1 μL 10 μM dNTP mix, 1 U DreamTaq (Fermentas) and 1 μL cDNA in a total volume of 25 μL, and was performed under the following conditions: 3 min 95 °C, 35 cycles (30 s 95 °C, 30 s 60 °C, 30 s 72 °C), 5 min 72 °C. PCR products were visualized by agarose gel electrophoresis.

### 4.7. Immunohistochemistry

To visualize macrophages and viral particles by immunohistochemistry (IHC), FRTs dissected from AF488-HPV16-PsVs (preincubated with BSA or SP-A) infected mice were prepared for cryosectioning. Briefly, tissues were fixed in 4% paraformaldehyde for 1 h, washed 3 × 10 min with PBS and suspended in 30% sucrose overnight at 4 °C. Tissues were embedded in Optimal Cutting Temperature (OCT, Sakura Finetec, Torrance, CA, USA) compound in a plastic mould (Sakura Finetec, Torrance, CA, USA) and frozen over dry ice. Tissue sections of 20 μm were cut using a Leica CM1850 cryostat, adhered to microscope slides coated with 3-Aminopropyltriethoxysilane (APES) and air dried overnight. For IHC staining, sections were fixed and permeabilised in methanol for 10 min at −20 °C, washed 3 × 10 min with PBS and blocked in 1% BSA for 1 h at room temperature. Macrophages were stained with a rat anti-mouse F4/80 antibody (CI:A3-1, Thermo Scientific) at a dilution of 1:1000 in 1% BSA blocking solution overnight at 4 °C in a sealed humidified chamber. Sections were washed 3 × 10 min with PBS and incubated with a goat anti-rat AF555 secondary antibody (Invitrogen, Carlsbad, CA, USA) at a 1:500 dilution in 1% BSA blocking solution for 90 min at room temperature. Following 3 × 10 min washes with PBS, sections were counterstained with 0.5 μg/ml Hoechst Nuclear Stain (Sigma) for 10 min at room temperature, washed in PBS, and mounted on glass slides with Mowiol (Sigma). Slides were visualized using a Zeiss LSM880 Airyscan confocal microscope together with Zen 2.3 SP1 software (Zeiss, Oberkochen, Germany). Epithelial structure was determined using the transmitted light and Hoechst channels only to eliminate bias. For quantification of macrophage recruitment, ten such areas were randomly selected for each condition, and Z-stacks were taken with the Hoechst, AF488 and AF555 channels selected. Z-stack dimensions were then normalised to 18 optical sections per image stack, and maximum intensity projections were done. The area occupied by macrophages (positive for F4/80) in the epithelium was determined by outlining the basal epithelium in each maximum intensity projection and determining the area occupied by the AF555 stain in these selected regions. This was normalized to the area occupied by the AF488-conjugated HPV16-PsVs, as the number of pseudovirions surrounding the basal epithelium is predicted to impact the level of immune response, i.e. the number of macrophages that are recruited to the basal epithelium. Normalizing was done for each individual maximum intensity projection as follows:

$$\frac{AF555\;\left(µm\;\times \;µ\times \right)}{AF488\;\left(µm\;\times \;µm\right)}\;\times \;100$$

The lower and upper thresholds were set at 18 and 256 greyscale levels, respectively, for each maximum intensity projection to remove background signal so that the measured signal for AF555 corresponded to the area representing F4/80 macrophage staining. 

### 4.8. Flow Cytometry Analysis of Genital Tract Immune Cell Populations

In order to prepare single cell suspensions, FRTs of uninfected mice was removed 6 h post N-9 treatment (Figure 5A), finely cut and digested in complete DMEM containing 1% HEPES and 20 µg/ml Liberase™ TL (Roche, Basel, Switzerland), for 1 h at 37 °C with gentle shaking. Digested tissue was passed through a 70 µm cell strainer and resuspended in fresh complete DMEM. Single cell suspensions were counted by trypan blue exclusion, using a haemocytometer slide and resuspended at a concentration of 1 × 10^7^ cells/ml. For time course experiments, 1 × 10^6^ cells were infected at 37 °C, 5% CO_2_ for 30 min to 6 h with 1 µg AF488-conjugated HPV16-PsVs which had been pre-incubated with purified human SP-A protein or BSA at a w/w ratio of 1:10 for 1 h at room temperature. At the indicated time points, innate immune cell populations were analysed by multi-colour flow cytometry. The following fluorochrome-conjugated antibodies were used: CD45 Alexa Fluor 700 (Biolegend, Clone: 30-F11, San Diego, CA, USA), CD11b Brilliant Violet (BV) 421™ (Biolegend, Clone: M1/70), SigLecF PE (Biolegend, Clone: S17007L), Gr-1 (Ly-6G+Ly-6C) APC Cy7 (eBioscience, Clone: RB6-8C5, San Diego, CA, USA), F4/80 BV605 (Biolegend, Clone: BM8). Cells were stained in staining buffer (0.5% BSA, 2 mM EDTA in PBS) for 20 min at 4 °C (dark), washed and resuspended in staining buffer for acquisition using the BD LSR Fortessa. Data was analysed using FlowJo software (V10). Innate immune cell populations were identified as CD45^+^CD11b^+^/SigLecF^+^/SSC^high^ (eosinophils), CD45^+^CD11b^+^/Gr-1^high^ (neutrophils), CD45^+^CD11b^+^/Gr-1^intermediate^ (monocytes) and CD45^+^CD11b^+^/F4/80^+^ (macrophages) (Supplementary Figure S1). Uptake of internalized AF488-labelled virions by immune cells were defined as AF488^+^ cells, based on fluorescence minus one (FMO) controls. 

## 5. Conclusions

This study is the first to demonstrate that SP-A but not SP-D binds directly to HPV16-PsVs and increases viral uptake into innate immune cells. This enhanced immune recognition leads to an overall decrease of cervicovaginal HPV infection in a murine model. The potentially protective functions of SP-A for genital HPV infection hold great promise for the development topical microbicides as alternative means against new HPV infections.

## 6. Patents

A patent application has been filed and is currently at PCT stage, pending national phase entry.

## Supplementary Materials

The following are available online at https://www.mdpi.com/2076-0817/8/4/288/s1, Figure S1: HPV+SP-A ex vivo infection: Innate cell gating strategy.
