# Supplementary figures and images for: Surfactant Protein A Impairs Genital HPV16 Pseudovirus Infection by Innate Immune Cell Activation in A Murine Model
Source: Pathogens. 2019 Dec 6;8(4):288. doi: 10.3390/pathogens8040288 (PMC6963799; doi:10.3390/pathogens8040288)

Suppl. Figure 1

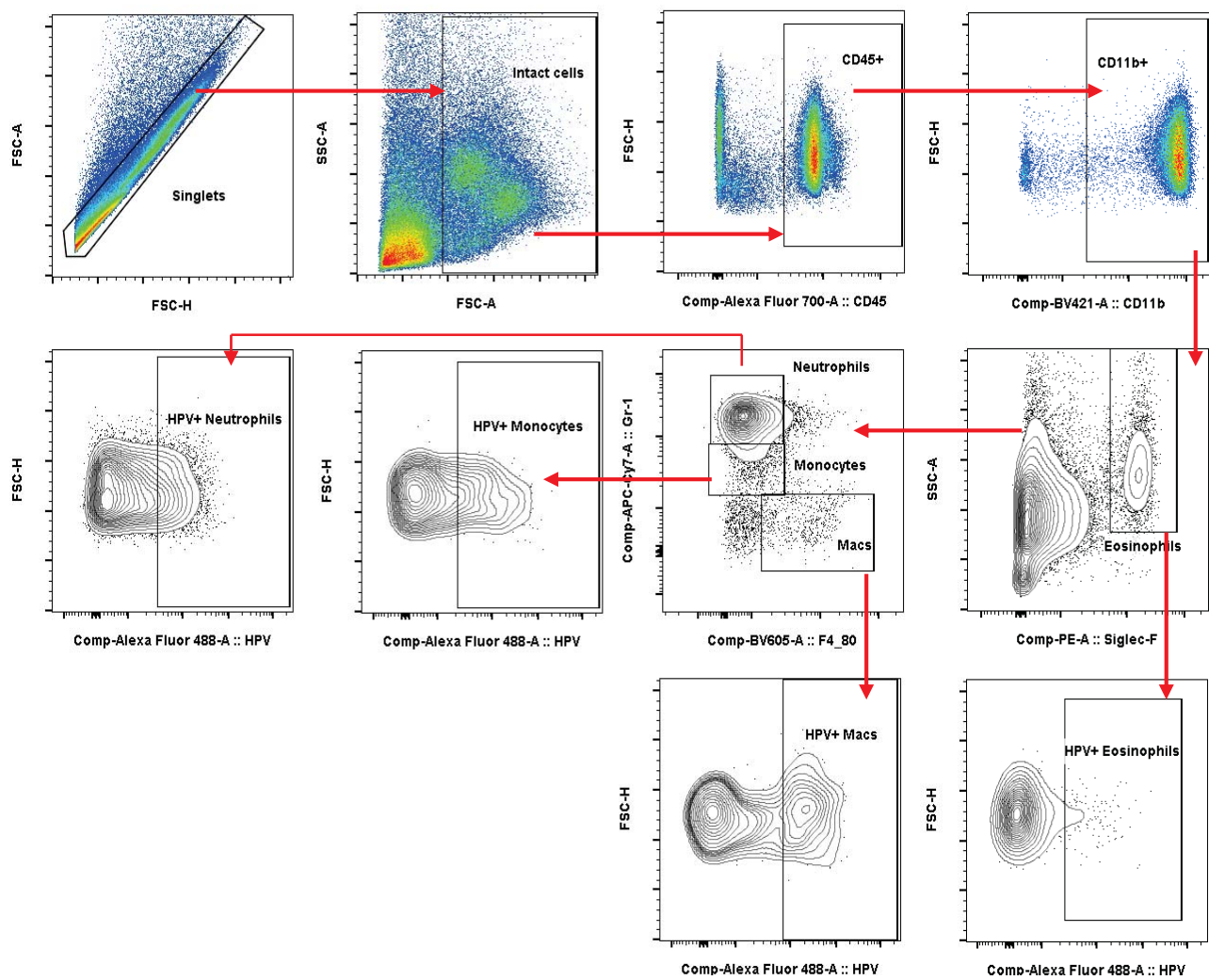

Supplement: Supplementary file 1 [file pathogens-08-00288-s001.pdf]
